# Impact of 5-HT4 Receptors on Neuron–Glial Network Activity In Vitro

**DOI:** 10.3390/ijms26167718

**Published:** 2025-08-09

**Authors:** Elena V. Mitroshina, Ekaterina A. Marasanova, Maria V. Vedunova

**Affiliations:** Institute of Biology and Biomedicine, Lobachevsky State University of Nizhny Novgorod, 23 Gagarin Avenue, 603022 Nizhny Novgorod, Russia; marasanova-2000-k@yandex.ru (E.A.M.);

**Keywords:** 5-HT4 receptor, serotonin, calcium activity, neuron–glial network, synapses

## Abstract

5-HT4 receptors play an important role in the regulation of synaptic plasticity. However, the effect of 5-HT4Rs on neural network activity and intercellular calcium signaling remains enigmatic. Using calcium imaging and original software, we determined the network-level characteristics of calcium dynamics within primary hippocampal cultures. We found that the single activation of 5-HT4 receptors by BIMU8 significantly reduced the correlation of activity within neuron–glial networks of primary cultures, without altering the proportion of active cells or the frequency of calcium events. In contrast, chronic stimulation of 5-HT4Rs promoted greater cell involvement in Ca^2+^ signal generation and increased the frequency of calcium events, while maintaining the connectivity level of the neuron–glial network. Moreover, our immunocytochemical labeling results indicated that chronic stimulation of 5-HT4Rs increased the size of both presynaptic and postsynaptic terminals. The acute blockade of 5-HT4Rs by RS23597-190 exerted a marked inhibitory effect on calcium activity in primary hippocampal cultures. Network connectivity and correlation of calcium activity were disrupted, and the number of functional connections among cells sharply declined. Our study showed that 5-HT4 receptors exhibit diverse effects based on the type and duration of activation, mediating several key functions in regulating neural network calcium activity.

## 1. Introduction

Serotonin (5-HT, 5-hydroxytryptamine) is widely recognized as a pivotal neurotransmitter in the central nervous system, modulating an extensive array of physiological processes, including mood, sleep, appetite, learning, and memory [1]. The breadth of serotonin’s functions is facilitated by its interactions with 5-HT receptors. Currently, at least 15 distinct subtypes of serotonin receptors are known, and they are grouped into seven families based on structural and functional characteristics [2,3]. Imbalances in serotonergic transmission are linked to numerous pathophysiological conditions, such as depression, obsessive–compulsive disorder, anxiety disorders, attention deficit hyperactivity disorder, autism spectrum disorders, and schizophrenia [4].

In recent years, particular focus has been placed on serotonin receptor 4 (5-HT4R) and its involvement in depressive, anxiety, cognitive, and other neuropsychiatric disorders [5,6,7]. In a healthy brain, 5-HT4 receptors play a role in regulating learning, memory, mood, nociception, and eating behaviors [4,8]. Consequently, dysfunction within the 5-HT4 receptor system may be implicated in related neuropsychiatric disorders, including prominent conditions such as depressive disorders and Alzheimer’s disease [4].

5-HT4Rs are part of the G-protein coupled receptor (GPCR) family, specifically Gαs-coupled, and they activate various adenylate cyclases. This activation generates cAMP, an intracellular second messenger that interacts with multiple targets, including protein kinase A and cyclic nucleotide-gated ion channels, resulting in modulation of calcium ion flux and changes in membrane excitability [5,9]. This function underscores the crucial role of 5-HT4Rs in regulating nerve impulse transmission and synaptic plasticity, as evidenced by multiple studies [10,11]. The research in [12] suggests that 5-HT4 mediates the modulation of calcium signaling in neuronal dendrites; however, comprehensive studies on the effect of 5-HT4Rs on neural network calcium activity and intercellular calcium signaling remain lacking. Calcium dynamics (calcium activity) refer to changes in the concentration of free Ca^2+^ ions in the cytoplasm of various neuronal and glial compartments. Ca^2+^ is a key second messenger in both neurons and glial cells, playing a central role in regulating numerous molecular cascades that govern essential functions of neurons and astrocytes. It is also critically involved in intercellular signaling. Disruptions in Ca^2+^ signaling are considered a hallmark of many neurodegenerative diseases.

It is also well-established that serotonin receptors influence neuronal morphology and synaptogenesis, which are critical factors for neural network functionality and the regulation of network activity. For instance, activation of 5-HT4Rs in hippocampal neurons has been shown to promote dendritic spine maturation and reduce neurite length and number through the Gα13/RhoA signaling pathway [13,14]. Nonetheless, this body of information remains limited, somewhat contradictory, and warrants further investigation.

Thus, the objective of this study was to investigate the role of 5-HT4Rs in modulating intercellular neuronal Ca^2+^ signaling.

## 2. Results

### 2.1. Effect of 5-HT4R Activation on Network Calcium Dynamics in Primary Neuronal Cultures

**Figure 1:**
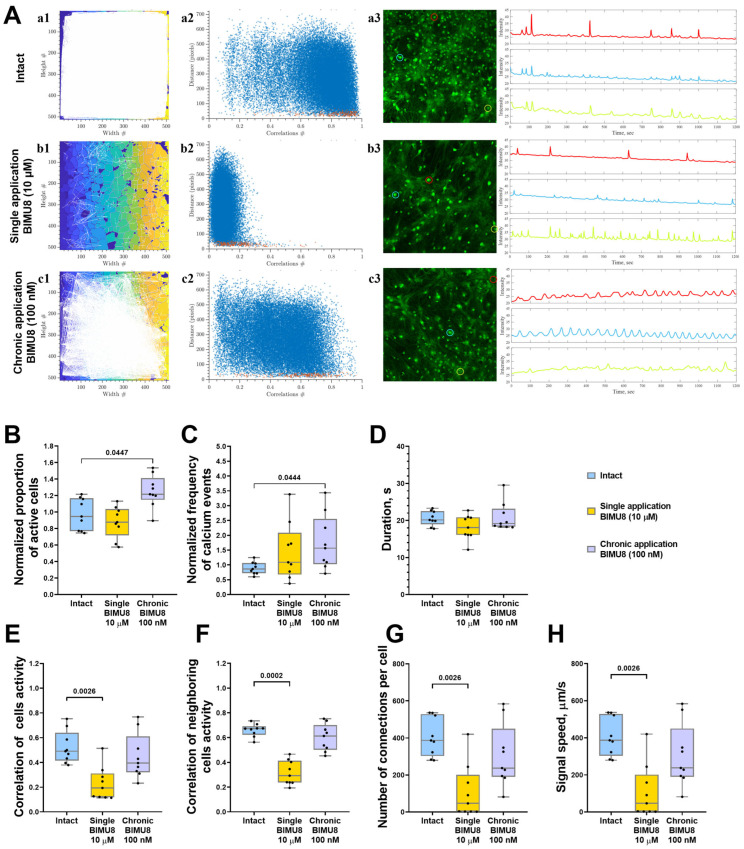
Analysis of calcium activity in primary hippocampal cell cultures upon activation of 5-HT4Rs by the selective agonist BIMU8. (**A**) Visualization of neuron–glial networks: (**a1**–**c1**)-representative directed graph examples visualizing the functional architecture of the neuron–glial network upon activation of 5-HT4Rs. Colored regions indicate the location of cell bodies, while white lines denote functional connections. Cells are considered functionally connected if their calcium activity correlation level exceeds a threshold of 0.3. (**a2**–**c2**) Representative scattergrams (point clouds) illustrating the correlation degree of calcium dynamics between cell pairs relative to the distance between them. In the scattergrams, red dots represent neighboring (directly contacting) cells, while blue dots indicate cells located further apart. (**a3**–**c3**) Representative examples of the dynamics of Oregon Green calcium sensor fluorescence in primary hippocampal cultures in vitro. Regions of interest (ROIs), corresponding to the cell soma, are marked by multi-colored circles. The change in fluorescence intensity of the Oregon Green calcium sensor within each ROI is represented by a curve of the corresponding color. (**B**) The proportion of cells exhibiting activity, normalized relative to the intact group. (**C**) The frequency of calcium events, normalized relative to the intact group. (**D**) The duration of calcium events, s. (**E**) The correlation of calcium activity between all pairs of cells in the field of view. (**F**) The correlation of calcium activity between neighboring pairs of cells in the field of view, (**G**) The number of functional connections per cell. (**H**) The speed of calcium signal propagation, µm/s. Statistically significant differences at *p* < 0.05 are represented with *p*-values; otherwise, a non-significant difference is recorded. Comparisons were made using the Kruskal–Wallis test.

We examined both individual calcium event characteristics—specifically, event duration, frequency, and the proportion of cells in which calcium events were recorded—and the network-level characteristics of calcium activity within primary neuronal cultures. By using the AstroLab original software program [15], we analyzed the correlation coefficients between calcium dynamics in cell pairs, focusing on both all cell pairs within the field of view and specifically on directly adjacent “neighboring” cells. We also assessed the rate of signal propagation between cells and quantified the number of functional connections within the cultures. A cell pair was classified as functionally connected if their calcium concentration dynamics showed a correlation coefficient above 0.3 [15]. This analytical approach enables the visualization of the functional architecture of neuron–glial networks through directed graph construction, facilitating the evaluation of network reorganization in response to pharmacological modulation of serotonin receptors (Figure 1A). Examples of the representative video of raw calcium activity primary hippocampal cultures are given in Supplementary Videos S1–S5.

In hippocampal neuron–glial networks, acute activation of 5-HT4 receptors using the BIMU8 agonist at a concentration of 10 μM negatively impacted network characteristics of calcium dynamics in primary cultures, leading to decreased connectivity within the neuron–glial network. A significant reduction was observed in the correlation of activity among both neighboring and all cells in neuron–glial networks (“Intact” 0.49 [0.41; 0.64], “Single BIMU8” 0.19 [0.12; 0.31]), alongside a significant decrease in the number of functionally significant connections between cells (“Intact” 386.8 [303.2; 528.1], “Single BIMU8” 46.55 [2.17; 201.5]) (Figure 1E–G). Although single acute exposure to BIMU8 did not alter the proportion of active cells displaying calcium events, it did reduce the signal propagation speed (“Intact” 386.8 [303.1; 528.1] µm/s; “Single BIMU8” 46.55 [2.16; 201.5] s) (Figure 1B,H).

Conversely, chronic 5-HT4 receptor activation via BIMU8 agonist at a concentration of 100 nM increased both the proportion of cells engaged in calcium activity generation (by 1.28 times) and the frequency of calcium events (by 1.81 times) (Figure 1B,C). However, it did not significantly impact the overall correlation of calcium dynamics across the cell culture (“Intact” 0.49 [0.41; 0.64]; “Chronic BIMU8” 0.40 [0.32; 0.61]) (Figure 1E,F).

**Figure 2:**
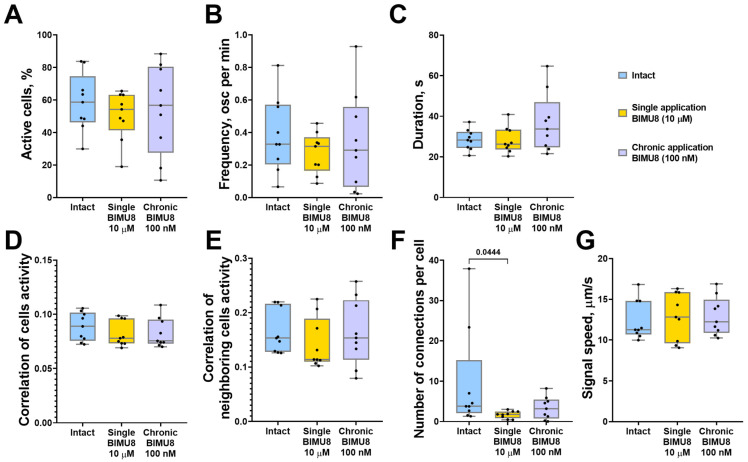
The main parameters of calcium activity in monoastrocytic mouse brain cell cultures in vitro upon activation of 5-HT4Rs by the selective agonist BIMU8. (**A**) The proportion of cells exhibiting activity, normalized relative to the intact group. (**B**) The frequency of calcium events, normalized relative to the intact group. (**C**) The duration of calcium events, s. (**D**) The correlation of calcium activity between all pairs of cells in the field of view. (**E**) The correlation of calcium activity between neighboring pairs of cells in the field of view. (**F**) The number of functional connections per cell. (**G**) The speed of calcium signal propagation, µm/s. Statistically significant differences at *p* < 0.05 are represented with *p*-values; otherwise, a non-significant difference is recorded. Comparisons were made using the Kruskal–Wallis test.

We also examined the role of 5-HT4Rs in regulating calcium dynamics within astrocytic networks in monoastrocytic cultures. Although astrocytes are electrically non-excitable, they are capable of generating and propagating Ca^2+^ signals, known as “calcium waves,” from cell to cell. Our previous work demonstrated that astrocytes can respond to external stimuli independently of neural afferentation, exhibiting a coordinated network response [15,16]. Given that 5-HT4Rs are present not only on neurons but also on astrocytic cells [5], studying the effects of 5-HT4Rs on collective dynamics of astrocyte calcium activity, isolated from the influence of neuronal signaling, is of particular interest. The analysis of calcium activity in glial networks revealed that both chronic and acute activation of 5-HT4Rs by the BIMU8 agonist did not significantly alter most of the calcium dynamic parameters in astrocytes (Figure 2). Representative videos of raw calcium activity of astrocytes are given in Supplementary Video S6–S10. However, acute single stimulation resulted in a significant decrease in the number of functional connections between cells (Figure 2F). However, despite the lack of statistically significant differences between groups in other parameters, astrocyte cultures exhibited a similar trend of reduced correlation between neighboring cells, as observed in neuron–glial networks (“Intact Astro” 0.15 [0.13; 0.22]; “Single BIMU8 Astro” 0.12 [0.11; 0.19]) during a single stimulation with the 5-HT4Rs agonist BIMU8 at a concentration of 10 μM (Figure 2E).

### 2.2. Effect of 5-HT4Rs Blockade on Network Calcium Dynamics in Primary Neuronal Cultures

**Figure 3:**
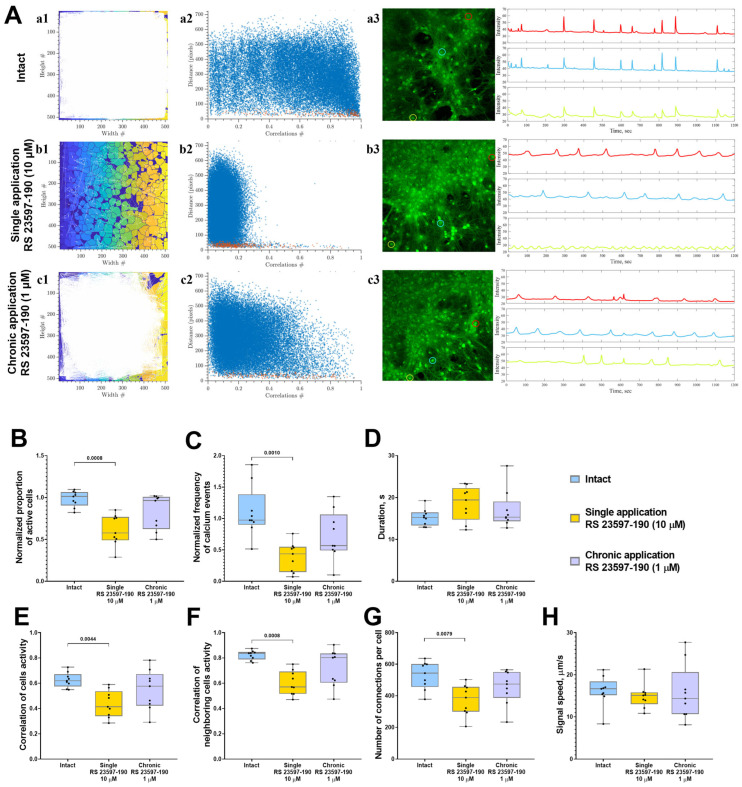
The analysis of calcium activity in primary hippocampal cell cultures in vitro upon the blockade of 5-HT4Rs with the selective antagonist RS 23597-190. (**A**) Visualization of neuron–glial networks: (**a1–c1**) Representative directed graph examples visualizing the functional architecture of the neuron–glial network upon activation of 5-HT4Rs. Colored regions indicate the location of cell bodies, while white lines denote functional connections. Cells are considered functionally connected if their calcium activity correlation level exceeds a threshold of 0.3. (**a2**–**c2**) Representative scattergrams (point clouds) illustrating the correlation degree of calcium dynamics between cell pairs relative to the distance between them. In the scattergrams, red dots represent neighboring (directly contacting) cells, while blue dots indicate cells located further apart. (**a3**–**c3**) Representative examples of the dynamics of Oregon Green calcium sensor fluorescence in primary hippocampal cultures in vitro. Regions of interest (ROIs), corresponding to the cell soma, are marked by multi-colored circles. The change in fluorescence intensity of the Oregon Green calcium sensor within each ROI is represented by a curve of the corresponding color. (**B**) The proportion of cells exhibiting activity, normalized relative to the intact group. (**C**) The frequency of calcium events, normalized relative to the intact group. (**D**) The duration of calcium events, s. (**E**) The correlation of calcium activity between all pairs of cells in the field of view. (**F**) The correlation of calcium activity between neighboring pairs of cells in the field of view. (**G**) The number of functional connections per cell. (**H**) The speed of calcium signal propagation, µm/s. Statistically significant differences at *p* < 0.05 are represented with *p*-values; otherwise, a non-significant difference is recorded. Comparisons were made using the Kruskal–Wallis test.

The investigation of calcium dynamics within neuron–glial networks using the selective 5-HT4 receptor antagonist RS 23597-190 revealed that chronic antagonist administration at a concentration of 1 μM did not produce significant changes in the key aspects of calcium dynamics or in the restructuring of functional network architecture. However, there was a tendency toward a reduction in the number of cells involved in calcium activity, the frequency of calcium events, and the number of functionally significant connections (Figure 3).

In contrast, a single application of RS 23597-190 at a concentration of 10 μM led to a notable inhibition of calcium activity, evidenced by a reduction in both the proportion of active cells generating calcium signals (decreased 1.7-fold-“Intact” 96.02 [81.36; 98.31]%; “Single RS 23597-190” 57.74 [38.26; 76.26]%) (Figure 3B) and the frequency of calcium events (decreased 2.9-fold: “Intact” 1.04 [0.85; 1.46] event/min; “Single RS 23597-190” 0.35 [0.21; 0.84] event/min) (Figure 3C).

Additionally, a statistically significant decrease in the correlation level of calcium dynamics within neuron–glial networks was observed (“Intact” 0.62 [0.57; 0.67]; “Single RS 23597-190” 0.41 [0.34; 0.54]) (Figure 3E). This was accompanied by a decrease in the amount of functional connections between cells (“Intact” 543.1 [456.1; 601.5]; “Single RS 23597-190” 388.4 [298.9; 456.9]; “Chronic RS 23597-190” 474.1 [386.1; 550.9]) (Figure 3G).

**Figure 4:**
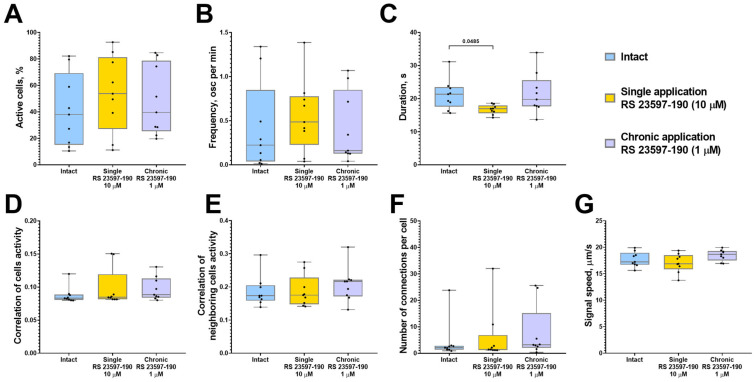
The main parameters of calcium activity in astrocytic networks of monoastrocytic cultures in vitro upon the blockade of 5-HT4Rs with the selective antagonist RS 23597-190. (**A**) The proportion of cells exhibiting activity, normalized relative to the intact group. (**B**) The frequency of calcium events, normalized relative to the intact group. (**C**) The duration of calcium events, s. (**D**) The correlation of calcium activity between all pairs of cells in the field of view. (**E**) The correlation of calcium activity between neighboring pairs of cells in the field of view. (**F**) The number of functional connections per cell. (**G**) The speed of calcium signal propagation, µm/s. Statistically significant differences at *p* < 0.05 are represented with *p*-values; otherwise, a non-significant difference is recorded. Comparisons were made using the Kruskal–Wallis test.

The analysis of calcium activity in astrocytic networks revealed that a single application of RS 23597-190 significantly reduced the duration of calcium events (“Intact” 21.34 [17.56; 23.47] s; “Single RS 23597-190” 16.95 [15.59; 17.95] s) but did not significantly impact other network parameters of calcium dynamics in astrocytes (Figure 4). The chronic blockade of 5-HT4 receptors did not result in any significant changes in calcium activity across the studied parameters.

**Figure 5:**
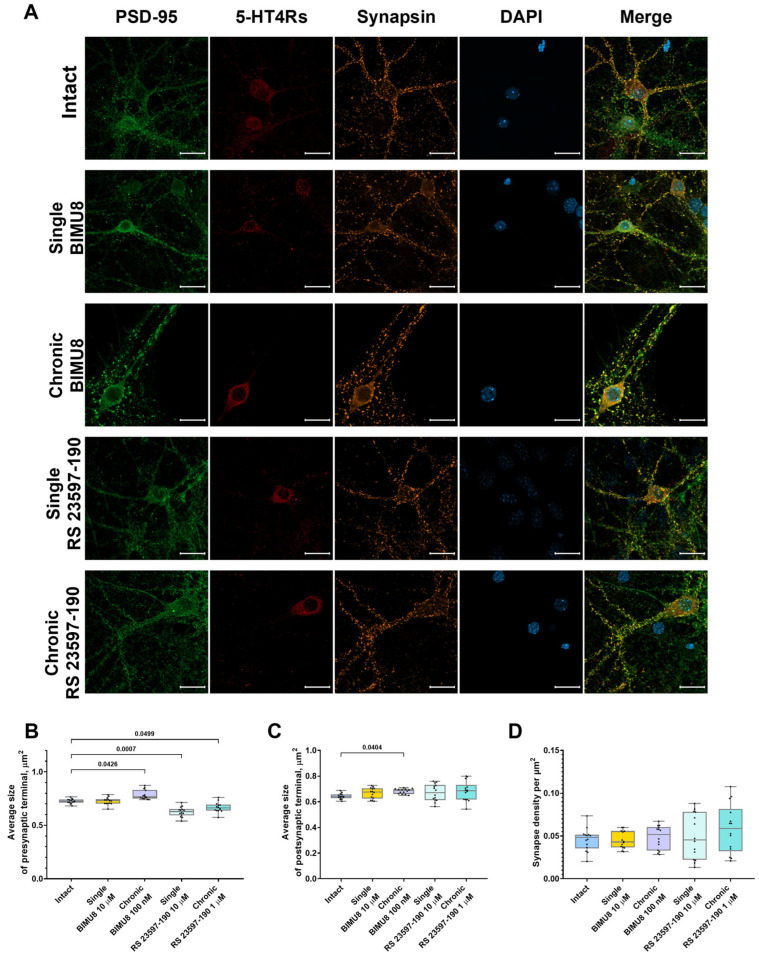
Impact of 5-HT4 receptor activity modulation on synaptic connections in hippocampal primary cell cultures**.** (**A**) Representative confocal images of immunocytochemical staining in primary hippocampal cell cultures at 14 DIV upon activation of 5-HT4Rs by the selective agonist BIMU8 and upon blockade of 5-HT4Rs by the selective antagonist RS 23597-190. Images were captured with a Plan-Apochromat 63x/1.40 Oil objective; the green channel shows postsynaptic region marker fluorescence (PSD-95), the orange channel shows presynaptic region marker fluorescence (synapsin), the red channel shows 5-HT4Rs marker fluorescence (SR-4), and the blue channel shows nuclear marker fluorescence (DAPI). Scale-20 µm. (**B**) Average size of presynaptic terminals, µm^2^. (**C**) Average size of postsynaptic terminals, µm^2^. (**D**) Synapse density per µm^2^. Statistically significant differences at *p* < 0.05 are represented with *p*-values; otherwise, a non-significant difference is recorded. Comparisons were made using the Kruskal–Wallis test.

**Figure 6:**
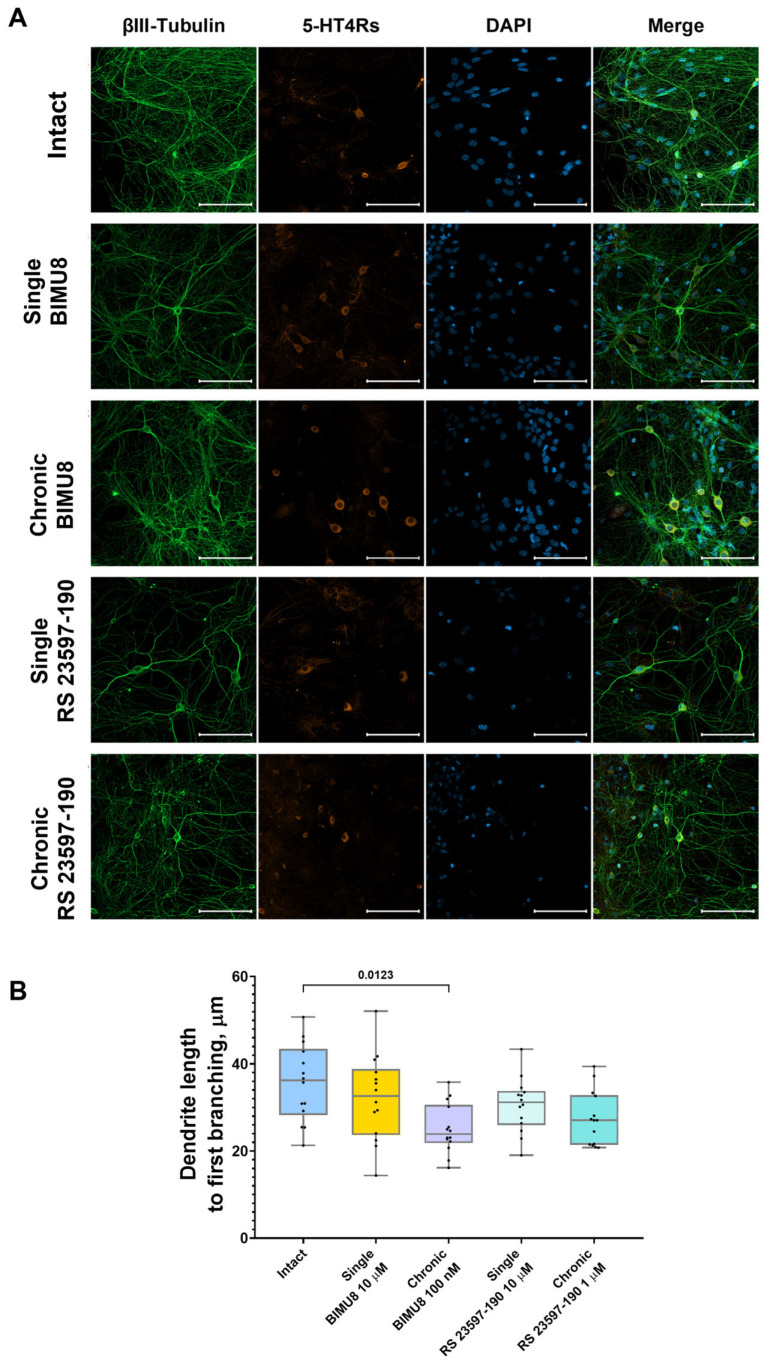
Impact of 5-HT4 receptor activity modulation on neuronal process morphology in hippocampal primary cell cultures (**A**) Representative confocal images of immunocytochemical staining in primary hippocampal cell cultures at 14 DIV. Images were captured with a Plan-Apochromat 20x/0.8 objective; the green channel shows neuronal marker fluorescence (βIII-Tubulin), the orange channel shows 5-HT4Rs marker fluorescence (SR-4), and the blue channel shows nuclear marker fluorescence (DAPI). Scale–100 µm. (**B**) Dendrite length to first branching. Statistically significant differences at *p* < 0.05 are represented with *p*-values; otherwise, a non-significant difference is recorded. Comparisons were made using the Kruskal–Wallis test.

One possible mechanism underlying the effect of 5-HT4Rs on neural network activity involves the regulation of neuron morphology and the maturation of synaptic contacts. To investigate this, we conducted the immunocytochemical labeling of neurites, pre-and postsynaptic terminals (Figure 5A and Figure 6A).

Analysis of confocal images from immunocytochemically stained primary hippocampal cultures at 14 DIV revealed that the chronic activation of 5-HT4 receptors results in a significant increase in the size of presynaptic terminals (“Intact” 0.72 [0.71; 0.74] μm^2^; “Chronic BIMU8” 0.77 [0.75; 0.83] μm^2^) (Figure 5B) and dendritic spines (“Intact” 0.64 [0.63; 0.66] μm^2^; “Chronic BIMU8” 0.69 [0.66; 0.70] μm^2^) (Figure 5C). Importantly, the density of synapses in the field of view remained unchanged (“Intact” 0.05 [0.04; 0.05] per µm^2^; “Chronic BIMU8” 0.05 [0.03; 0.06] per µm^2^) (Figure 5D). Furthermore, a statistically significant reduction in dendrite length from the cell body to the first branching point was observed (“Intact” 36.21 [28.28; 43.46] μm; “Chronic BIMU8” 23.88 [21.86; 30.63] μm) (Figure 6B).

In contrast, acute stimulation of 5-HT4 receptors with the selective agonist BIMU8 did not have a pronounced effect on the examined aspects of neuronal morphogenesis or synaptogenesis (Figure 5 and Figure 6).

Neither the acute nor chronic blockade of 5-HT4 receptors with the antagonist RS 23597-190 resulted in changes to the dendrite length prior to the first branching point (“Intact” 36.21 [28.28; 43.46] μm; “Single RS 23597-190” 31.20 [25.96; 33.79] μm; “Chronic RS 23597-190” 27.08 [21.45; 32.82] μm) (Figure 6B). Additionally, the blockade did not affect the size of the postsynaptic region (“Intact” 0.64 [0.63; 0.66] μm^2^; “Single RS 23597-190” 0.67 [0.61; 0.73] μm^2^; “Chronic RS 23597-190” 0.69 [0.62; 0.73] μm^2^) (Figure 5C) or the density of synapses in the field of view (“Intact” 0.05 [0.04; 0.05] per µm^2^; “Single RS 23597-190” 0.05 [0.02; 0.08] per µm^2^; “Chronic RS 23597-190” 0.06 [0.03; 0.08] per µm^2^) (Figure 5D). However, both acute and chronic blockade caused a significant reduction in the average pre-synapse size (“Intact” 0.72 [0.71; 0.74] μm; “Single RS 23597-190” 0.63 [0.59; 0.65] μm^2^; “Chronic RS 23597-190” 0.66 [0.64; 0.69] μm^2^) (Figure 5B).

## 3. Discussion

5-HT4 receptors are among the relatively less explored types of serotonin receptors. Known to play an essential role in modulating neuronal signaling and synaptic plasticity, they are also critical in learning and memory processes and act as regulators of synaptogenesis. This makes investigating their influence on the regulation of functional activity and structural architecture within neuron–glial networks particularly significant.

In our study, we examined the effects of 5-HT4 receptor modulation on the functional calcium activity of neuron–glial and astrocytic networks and on neuronal morphogenesis in vitro. A single exposure to BIMU8 resulted in a 2.44-fold decrease in the correlation level of calcium dynamics in cells of primary hippocampal cultures and a 3.8-fold reduction in the number of functionally significant connections. Importantly, the number of active cells did not decrease, nor did the frequency of calcium events. This suggests that acute activation of 5-HT4Rs induces desynchronization in the activity of nerve cells within the neuron–glial network, which may subsequently lead to network restructuring and reconsolidation. Indeed, during chronic stimulation, the connectivity of the neuron–glial network is comparable to that of the intact group. However, more cells participate in network activity (the proportion of active cells increases by 12%), and their calcium activity is enhanced, with the frequency of calcium events increasing by 1.58 times. Thus, chronic activation of 5-HT4Rs elevates the baseline level of calcium neural network dynamics without disrupting the formation of functional connections between cells.

In contrast, acute blockade of 5-HT4Rs results in a significant reduction in neural network calcium activity. The number of cells involved in calcium dynamics decreases by more than 1.5 times, the number of functional connections drops by 1.42 times, and the correlation coefficient between both distant and neighboring cells is significantly reduced. This effect likely stems from the abrupt closure of cAMP-dependent calcium channels due to 5-HT4 receptor inactivation and subsequent reduction in cAMP synthesis.

These findings underscore the critical role of 5-HT4Rs in maintaining neural network interactions. However, during chronic blockade, these disruptions are not observed, suggesting the presence of compensatory mechanisms that help balance calcium activity independently of 5-HT4 receptors.

We also investigated the effects of 5-HT4R modulation on astrocytic network calcium signaling in the absence of neuronal regulation, assessing the impact of a 5-HT4R agonist and antagonist on calcium dynamics in monoastrocytic cultures. Our findings indicated that neither acute nor chronic blockade or stimulation of 5-HT4Rs produced significant changes in calcium dynamics, with the sole exception being a reduction in functional connections under acute BIMU8 stimulation. This suggests that observed changes in calcium dynamics within neuron–glial networks are largely mediated by neuronal signaling.

Our data expand current knowledge regarding the role of 5-HT4Rs in neural network functioning and synaptic plasticity. 5-HT4 receptors are Gαs-coupled and facilitate the activation of adenylate cyclase. The cAMP produced during the adenylate cyclase reaction acts as an intracellular second messenger, interacting with various targets, including protein kinase A and cyclic nucleotide–gatedion channels. This interaction modulates calcium ion flux and alters membrane excitability [9,17]. These effects, resulting from changes in serotonin signaling via 5-HT4Rs, are expected to influence cytoplasmic calcium dynamics in nerve cells, as demonstrated in [12]. However, our study is the first to describe the effects of 5-HT4Rs on the functional calcium activity of neural networks.

The effects of chronic 5-HT4R modulation are likely of a different nature, associated with 5-HT4R’s role in synapse formation and maturation. Our immunocytochemical labeling results indicated that chronic stimulation of 5-HT4Rs, unlike acute stimulation, increased the size of both presynaptic and postsynaptic terminals and accelerated dendritic branching (with significantly shorter dendrite length before the first branching in cultures chronically exposed to BIMU8). Synapse growth and maturation are associated with an increase in the area and number of presynaptic active zones and postsynaptic densities. This leads to an increase in the number of voltage-gated calcium channels and increased calcium signaling. Both acute blockade and chronic blockade of 5-HT4Rs reduced presynaptic region size.

These findings align with previous studies. For example, functional loss of 5-HT4R due to knockout led to the dysregulation of genes essential for synaptic plasticity. In 5-HT4R knockout mice, an increase in immature neurons was observed in the ventral, but not dorsal, dentate gyrus, along with persistent Alzheimer’s disease-like behavioral deficits and heightened baseline anxiety [11]. Hashemi-Firouzi et al. in [18] investigated the effects of chronic 5-HT4R stimulation with BIMU8 on cognitive function, memory, long-term potentiation, paired-pulse ratio, and neuronal apoptosis in rats with amyloid beta (Aβ)-induced Alzheimer’s disease. BIMU8 facilitated LTP formation and improved cognitive function in animals, likely by reducing apoptotic hippocampal neurons and enhancing hippocampal synaptic functions. Moreover, 5-HT4 receptors have been implicated in synaptic contact maturation and dendritic growth regulation [19]. These effects of 5-HT4R may be associated with the cAMP-mediated activation of protein kinase A, followed by the activation of ERK through the Ras-Raf-MEK1/2 pathway, involving the MEK-mediated dual phosphorylation of ERK on threonine and tyrosine residues. Alternatively, ERK activation may occur via the interaction of cAMP with the Epac1/2 family (exchange proteins directly activated by cAMP), which are cAMP sensors. Extracellular-signal-regulated kinase (ERK), is an intermediate link in the MAPK activation cascade of the cAMP-sensitive transcription factor CREB (cAMP response element-binding protein), which induces the expression of brain-derived neurotrophic factor (BDNF) and other proteins crucial for neuronal differentiation, neurite outgrowth, and synaptogenesis [5].

It is important to acknowledge several limitations of this study. First, to evaluate the effects of 5-HT4Rs on inter-astrocytic calcium signaling, we utilized mono-astrocytic cultures. This approach demonstrated that neither activation nor blockade of 5-HT4Rs caused significant changes in the calcium dynamics of astrocytic networks in the absence of neuronal signaling. In future research, it would be valuable to conduct imaging experiments on neuron–glial hippocampal cultures using genetically encoded calcium sensors, such as GCaMP, expressed under cell population-specific promoters. This would enable selective analysis of calcium signals in astrocytes and neurons within neuron–glial networks, providing new insights into the impact of serotonin modulation on neural network functioning.

The stimulatory effect of chronic 5-HT4 receptor (5-HT4R) activation on synapse size and calcium activity in hippocampal cells may prove beneficial for restoring neuronal function lost in neurodegenerative processes and depressive disorders. Several studies highlight the involvement of 5-HT4Rs in mood disorders and other psychiatric conditions. For example, changes in 5-HT4R expression have been observed in rodent models of depression, though findings vary by model. Some studies report a decrease in 5-HT4R expression, while others find an increase [20,21]. Similarly, human studies have yielded conflicting results [22,23]. Additionally, different splice variants of the C-terminal domain of 5-HT4Rs are associated with a predisposition to unipolar depression [24]. Deletion or pharmacological blockade of 5-HT4Rs has been shown to increase depressive and anxiety-like behaviors in rodents [25,26,27,28,29,30]. Conversely, 5-HT4R agonists have shown efficacy as experimental antidepressants [31,32], increasing hippocampal BDNF mRNA expression [33]. Moreover, 5-HT4R agonists are thought to promote neurogenesis in the adult brain, enhancing neurogenesis in both the dentate gyrus and the enteric nervous system [32,33].

5-HT4Rs are also implicated in Alzheimer’s disease, where they play a role in interacting with the alpha-secretase ADAM10, promoting non-amyloidogenic cleavage of the amyloid precursor protein and thus preventing pathological amyloid plaque formation [34]. Agonist-induced activation of 5-HT4Rs has been shown to inhibit cognitive deficits by enhancing spatial learning and recognition abilities in rodents [35,36]. In the 5xFAD mouse model of Alzheimer’s disease, chronic activation of 5-HT4Rs by agonists significantly reduced cerebral astrogliosis and microgliosis, which are inflammatory processes associated with the progression of AD [37].

The growing body of experimental evidence highlighting the distinct effects of various 5-HT receptor subtypes underscores the potential of selective agonists and antagonists as candidates for next-generation antidepressants and other therapeutics for neurological disorders. Our findings suggest that the 5-HT4R agonist BIMU8 enhances neural network activity and synaptic maturation, offering potential applications in countering neurodegenerative processes, compensating for synapse and neuron loss, and ameliorating neural network dysfunction in depressive states. Continuing to study the effects of 5-HT4R modulation across various neurological pathology models appears promising for the development of effective therapeutic strategies.

## 4. Materials and Methods

All experimental protocols in this study were approved by the Bioethics Committee of Lobachevsky University and conducted in compliance with Law No. 708n (23.08.2010) of the Ministry of Health of the Russian Federation, which establishes laboratory practice standards for the care and use of laboratory animals.

### 4.1. Cultivation of Primary Mixed Hippocampal Cultures and Monoastrocytic Cultures

This study utilized primary hippocampal cell cultures derived from brains of C57BL/6 mouse embryos at gestational day 18 (E18). Cells were seeded onto 12-well culture plates for calcium imaging and onto 18 × 18 mm coverslips for immunohistochemical analysis.

Primary hippocampal cultures were prepared following the protocol outlined by Mitroshina et al. [16,38]. Embryonic brain was cleared of skin and cranial bones and immersed in PBS solution (ThermoFisher, Massachusetts, MA, USA). The hippocampi were then surgically isolated, mechanically dissociated, and incubated in trypsin for 20 min at 37 °C. After three PBS washes, cells were resuspended in Neurobasal Medium™ (ThermoFisher, USA) and seeded onto 12-well plates (the cell density ranged from 8500 to 9000 cells/mm^2^). The culture medium was supplemented with 0.5 mM L-glutamine (ThermoFisher, USA), 1% B27 supplement (ThermoFisher, USA), and 0.5% fetal bovine serum (Capricorn Scientific GmbH, Ebsdorfergrund, Germany) [16]. In previous work, we have repeatedly reported data on the cellular composition of primary hippocampal cell cultures obtained using our established protocol [38,39,40]. These results demonstrate that, at 21 DIV, primary hippocampal cultures contain both neurons and astrocytes in an approximate ratio of 1:2 [38]. Immunocytochemical labeling of the cellular composition of primary hippocampal cultures is presented in Figure 1 of the Supplementary Figure S1.

Astrocytic cultures were prepared from the hippocampus of neonatal C57BL/6 mice aged P1–P6. Mice were sacrificed by decapitation, and the hippocampi were surgically isolated, cleared of skin and cranial bones, and mechanically dissociated in a PBS solution (BioinnLabs, Rostov-on-Don, Russia). Astrocyte cultures were prepared from the cerebral cortex of neonatal C57BL/6 mice (P1–P6). Passaging was performed on day 7 of cultivation. Cells were detached from the substrate using a trypsin–versene solution (1:3) (PanEco, Moscow, Russia), followed by washing and centrifugation. The cells were then reseeded at a density of approximately 4500 cells/mm^2^. This protocol ensures the elimination of the neuronal population, as confirmed in our earlier work [16]. Immunocytochemical labeling of the cellular composition of primary astrocyte cultures is presented in Figure 2 of the Supplementary Figure S2. Suppression of microglia and oligodendrocyte growth was not carried out. The resulting cell suspension was resuspended in 1 mL of RPMI 1640 culture medium (BioinnLabs, Russia) supplemented with 0.5 mM L-glutamine (Gibco, Montana, MT, USA), 1% B27 (Gibco, USA), 10% FBS (Biowest, Nuaillé, France), and 1% sodium pyruvate (Gibco, USA). Cells were seeded into 12-well culture plates pre-treated with polyethyleneimine solution (Merck, Darmstadt, Germany) to enhance adhesion, at a density of 8500–9000 cells/mm^2^. After 40 min, once the cells had adhered to the substrate, 1 mL of growth medium was added [15,16].

The viability of primary cultures was maintained in a CO_2_ incubator set to 37 °C and 5% CO_2_ for a period of 14 or 21 days, with 50% of the culture medium replaced approximately every 48 h. The morphological state of the primary cultures was assessed during each medium replacement using an Axio Observer A1 inverted microscope (Zeiss, Jena, Germany).

### 4.2. Modulation of 5-HT4Rs Activity in Vitro

The selective agonist BIMU8 (Tocris, Bristol, UK) was used to activate 5-HT4Rs, while the selective 5-HT4 receptor antagonist RS 23597-190 (Sigma-Aldrich, Missouri, USA) was employed to block 5-HT4Rs.

Two protocols were implemented for 5-HT4Rs modulation, acute and chronic. Acute activation involved the application of BIMU8 at a concentration of 10 μM on the 14th day in vitro (DIV) for hippocampal cultures or on the 21st DIV for monoastrocytic cultures, administered 60 min before recording calcium activity or before fixing the cultures [41]. For acute 5-HT4Rs blockade, RS 23597-190 was applied at a concentration of 10 μM on the 14th DIV for hippocampal cultures or the 21st DIV for monoastrocytic cultures, 60 min prior to calcium activity recording or culture fixation.

For chronic 5-HT4Rs activation, 100 nM BIMU8 was added with each medium change from day 1 until the end of the culturing period [42]. For chronic 5-HT4Rs blockade, RS 23597-190 was introduced at a concentration of 1 μM [43], beginning at 1 DIV, with each medium change continued until the end of the culturing period.

### 4.3. Fluorescent Calcium Imaging for Assessing Functional Calcium Activity of Neuron–Glial and Astrocytic Networks

We used the calcium imaging technique for visualization of the functional organization of neural networks at the cellular level. It is one of the classical methods for examining metabolic activity within the entire neuron–glial network as well as within individual components, including neurons and glial cells [44,45].

Calcium intercellular signaling was recorded at 14 DIV for hippocampal neuron–glial cultures and at 21 DIV for astrocytic cultures. Oregon Green 488 BAPTA-1 AM (OGB-1), at a concentration of 0.4 μM (Invitrogen, USA), was dissolved in dimethyl sulfoxide (DMSO) (Merck KGaA, Darmstadt, Germany) containing 4% Pluronic F-127 (Thermo Fisher Scientific, Waltham, MA, USA), in accordance with the manufacturer’s recommendations, and used as a calcium-sensitive fluorescent indicator. Cultures were incubated with the dye for 45 min at 37 °C in a CO_2_ incubator. Zeiss LSM 800 confocal microscope (Carl Zeiss, Jena, Germany) was carried for visualization. To assess intracellular calcium dynamics, time-lapse image series were acquired with Plan-Apochromat 10x/0.3 objective, resolution of 512 × 512 pixels, and a field of view of 639 × 639 μm, at a frame rate of 2 Hz, for a total recording duration of 20 min. Fluorescence excitation was achieved using a 488 nm LED light source, and emission was recorded with a light filter allowing wavelengths between 500 and 565 nm.

Calcium event durations vary across cell types. Astrocytic calcium events typically last 15–20 s [15,46,47,48]. Events associated with interneuronal signaling that locally modulate intracellular calcium concentration exhibit millisecond temporal resolution. However, current calcium imaging methods cannot resolve such events within neuron–glial networks comprising dozens of active cells. At this resolution, network activity is assessed using large-scale calcium events that encompass entire cells and integrate tens to hundreds of concurrent local calcium events. These events can persist from several seconds [49,50] up to 1.5 min, as reported in certain pathological conditions [51]. In this study, we employed mathematical analysis to detect signal correlations within the network and map activity propagation profiles. This required high spatiotemporal resolution of cellular compartments. Considering both methodological objectives, an optimal calcium activity detection rate of 2 frames per second was implemented [15,16].

Data analysis was carried out using the custom software package “AstroLab” which employs an algorithm we developed to analyze network characteristics of calcium activity [certificates of state registration of software No. 2021612870 dated 25.02.2021; No. 2022667580 dated 22.09.2022; No. 2022667691 dated 23.09.2022; No. 2022668038 dated 29.09.2022] [15,16]. A range of parameters was evaluated, including calcium event duration, frequency, and the percentage of active cells in the culture (the proportion of cells exhibiting at least one calcium event relative to the total number of cells within the field of view).

Calcium events are defined as spontaneous, periodic increases in cytoplasmic calcium ion concentration that occur in response to various stimuli. A calcium event is characterized as a continuous period during which the calcium signal exceeds two exponential moving averages (EMA) of that signal. The calcium signal of a cell was defined as the mean fluorescence intensity of the calcium indicator across all pixels within the spatial region corresponding to the cell. Prior to analysis, a spatiotemporal denoising filter (BM3D) was applied to the signal.

The duration of the calcium event is defined as the time interval between the onset and termination of an event, calculated based on when the relative calcium signal exceeds the adaptive threshold.

To investigate neuron–glial and astrocytic network reorganization in response to 5-HT4R activation and blockade, network characteristics of calcium dynamics were assessed. Parameters included the degree of correlation in calcium activity between all pairs of cells in the culture and between adjacent (directly contacting) cells, the number of functional connections per cell (pairs of cells with significantly correlated calcium activity above a threshold value of 0.3 were considered functionally connected), and the propagation speed of the calcium signal [15]. To assess network connectivity, we used the correlation coefficient between the average calcium activity levels of individual cells. The correlation of calcium activity reflects the similarity of activity patterns and the degree of synchronization between cells. The proposed approach to constructing a dynamic neuron–glial network enables the identification of functional connections between cells, as well as the detection of network activity and its changes over time. The developed algorithm models the neuron–glial (or astrocytic) network as a directed graph, where vertices represent individual cells and edges denote above-threshold correlations between calcium events. A directed functional connection is inferred when the correlation between a pair of cells reaches a maximum with a significant temporal delay between their calcium signals. A functional connection was defined as a correlation between the processed calcium signals of two cells that exceeded the threshold value, accounting for a time lag of ±5 s. The methods for calculating the correlation coefficient, detailed in [15], are insensitive to changes in baseline calcium activity levels or the frequency of calcium events. Instead, variations in this coefficient reflect alterations in the connectivity of the functional dynamic network in response to various stimuli.

### 4.4. Immunocytochemical Staining of Primary Hippocampal Cultures

Prior to immunocytochemical staining, cultures grown on coverslips were fixed in a solution of 4% paraformaldehyde (PFA) and 15% sucrose. Fixation was conducted for 15 min in a CO_2_ incubator set to 37 °C and 5% CO_2_.

The immunocytochemical staining protocol included blocking non-specific binding for 1 h using a blocking buffer (5% FBS, 0.5% Triton X-100, 0.1% Tween 20 in 1x PBS). Primary antibody incubation followed, with a dilution of 1:500, for 3 h at +4 °C. The primary antibodies we used were mouse anti-ßIII-tubulin (neuron marker, ab7751, Abcam, UK); rabbit anti-SR-4 (5-HT4Rs marker, ES3494, ELK Biotechnology, USA); mouse anti-psd95 (post-synaptic terminal marker, ab192757, Abcam, UK); and chicken anti-synapsin (presynaptic terminal marker, Cat. No. 106 006, Synaptic Systems, Germany). After primary antibody incubation, cultures were washed three times with wash buffer (0.1% Triton X-100, 0.1% Tween 20 in 1x PBS). Treatment with secondary antibodies (1:1000 dilution) lasted 90 min. Secondary antibodies included anti-mouse Alexa488 (Invitrogen, USA), anti-chicken Alexa647 (Invitrogen, USA), anti-rabbit Alexa555 (Invitrogen, USA), and anti-rabbit Alexa647 (Invitrogen, USA). Following three additional washes with wash buffer, cultures were stained with DAPI (1:1000, 10 min) and mounted in fluoromount-G mounting medium (Invitrogen, USA).

Confocal images were captured using an LSM 800 confocal laser scanning microscope. For each culture, at least ten different fields of view were imaged. Image analysis was conducted using the open-source software ImageJ 1.54g, with the Synapse_Counter plugin employed to quantify the number of postsynaptic and presynaptic sites and their colocalization within each field of view and per unit length of dendrite. Synapses were defined as regions of colocalization between presynaptic and postsynaptic protein clusters, identified through immunocytochemical labeling. To minimize artifacts and ensure accurate quantification, we followed the parameter optimization protocol for the Analyze Particles function, as outlined by the plugin developers. The length of dendrites up to the first branching point was measured manually by tracing the segment from the neuron soma to the initial dendritic bifurcation. Only those dendritic processes were included for which the first branching could be unambiguously distinguished from overlapping neuronal projections.

### 4.5. Statistical Data Analysis

All experiments were conducted in a minimum of three biological replicates. To ensure robustness of the results, each biological replicate included three technical replicates, with calcium activity recorded in three distinct fields of view per replicate. For immunohistochemical analyses, at least 10 fields of view were examined for each of the three biological replicates (Table 1).

## 5. Conclusions

Our study demonstrates that 5-HT4 receptors exhibit diverse effects based on the type and duration of activation, mediating several key functions in regulating neural network activity. These findings underscore the importance of investigating 5-HT4Rs both in basic research and as potential therapeutic targets for treating neuropsychiatric disorders with significant societal impact.

## Figures and Tables

**Table 1:** Number of experimental animals and neural cell cultures used in the experiment.

Methodology	Number of Animals	Number of Cultures
Calcium imaging	18 E18 mouse embryos obtained from 6 different pregnant females for hippocampal cultures; 18 newborn P1–P3 mice for monoastrocytic cultures	Hippocampal cultures: 54; monoastrocytic cultures: 54. Three fields of view were analyzed per culture, with at least 150 neurons per field of view
Immunocytochemical studies	14 E18 mouse embryos obtained from 4 different pregnant females	Hippocampal cultures: 42. At least 10 fields of view were analyzed per culture. The average number of neurons analyzed per field of view was 2–3 at x63 magnification and 8–10 at x20 magnification

Statistical analysis was carried out using GraphPad Prism (v.9.2). For data following a normal distribution, analysis of variance (ANOVA) was applied. For data not conforming to a normal distribution, the nonparametric Kruskal-Wallis test was used. Data performed as median ± interquartile range (IQR). Statistical significance was determined at *p* < 0.05.

## Data Availability

The data used to support the findings of this study are available from the corresponding author upon request.

## Supplementary Materials

The following supporting information can be downloaded at: https://zenodo.org/records/16777806, accessed on 4 August 2025.

## Abbreviations

The following abbreviations are used in this manuscript:

5-HT	5-hydroxytryptamine, serotonin
5-HTRs	Serotonin receptors
cAMP	Cyclic adenosine monophosphate
DIV	Days in vitro
E18	Gestational day 18
ERK	Extracellular signal-regulated kinase
GPCR	G-protein coupled receptor
IQR	Interquartile range
MAPK	Mitogen-activated protein kinase
P1	Postnatal day 1
